# Spontaneous Versus Controlled Mechanical Ventilation in Patients with Acute Respiratory Distress Syndrome

**DOI:** 10.1007/s40140-021-00443-8

**Published:** 2021-03-03

**Authors:** Tayyba Naz Aslam, Thomas Lass Klitgaard, Kristin Hofsø, Bodil Steen Rasmussen, Jon Henrik Laake

**Affiliations:** 1 Department of Anaesthesiology, Division of Emergencies and Critical Care, Rikshospitalet Medical Centre, Oslo University Hospital, P.O.Box 4950, Nydalen, Oslo, Norway; 2 Department of Research and Development, Division of Emergencies and Critical Care, Oslo University Hospital, Oslo, Norway; 3 Department of Anaesthesia and Intensive Care Medicine, Aalborg University Hospital, Aalborg, Denmark; 4 Department of Clinical Medicine, Aalborg University, Aalborg, Denmark; 5 Lovisenberg Diaconal University College, Oslo, Norway

**Keywords:** Acute respiratory distress syndrome, Mechanical ventilation, Spontaneous ventilation, ARDS, Acute lung injury, ALI

## Abstract

**Purpose of Review:**

To review clinical evidence on whether or not to allow mechanically ventilated patients with acute respiratory distress syndrome (ARDS) to breathe spontaneously.

**Recent Findings:**

Observational data (LUNG SAFE study) indicate that mechanical ventilation allowing for spontaneous breathing (SB) is associated with more ventilator-free days and a shorter stay in the intensive care unit without any effect on hospital mortality. A paediatric trial, comparing airway pressure release ventilation (APRV) and low-tidal volume ventilation, showed an increase in mortality in the APRV group. Conversely, in an unpublished trial comparing SB and controlled ventilation (NCT01862016), the authors concluded that SB is feasible but did not improve outcomes in ARDS patients.

**Summary:**

A paucity of clinical trial data continues to prevent firm guidance on if or when to allow SB during mechanical ventilation in patients with ARDS. No published large randomised controlled trial exists to inform practice about the benefits and harms of either mode.

## Introduction

Acute hypoxaemic respiratory failure (AHRF) may progress to acute respiratory distress syndrome (ARDS), a common but underrecognized cause of admission to the intensive care unit (ICU), that is highly lethal and associated with significant morbidity in survivors [1, 2••, 3, 4]. There are no specific therapies for ARDS, and invasive mechanical ventilation remains a mainstay in the management of these patients [5]. Over the last 20 years, a number of randomised clinical trials have improved our understanding of how patients with ARDS can best be managed, with the potential for a large reduction in mortality [6].

Current guidelines stress the importance of early recognition of ARDS in patients with AHRF and evidence-based practices in the management of these patients [7–9]. Such practices include:

All ARDS patients:

1. Pressure and volume limitation in mechanical ventilation (strong recommendation).
2. Fluid restriction (weak recommendation).

Patients with moderate to severe ARDS [1]:

3. Higher positive end-expiratory pressure (PEEP) (weak recommendation).
4. Recruitment manoeuvres (weak recommendation).
5. Ventilation of patients in the prone position (weak recommendation).
6. Utilisation of neuromuscular blocking agents (NMBA) (weak recommendation).

Guideline developers were unable to provide evidence-based recommendations for several clinically relevant questions, including ventilator mode (volume-controlled vs. pressure-controlled ventilation, modes allowing spontaneous breathing (SB), i.e. triggered by the patient, vs. fully controlled ventilation), or fraction of inspired oxygen (FiO_2_) or oxygenation target, due to lack of data from clinical trials [7–9].

Intensivists worldwide have divided opinions on whether SB during mechanical ventilation in ARDS patients is harmful or not. This paper is a narrative review of recent evidence divided into two sections according to the following statements:

I. Spontaneous breathing efforts during mechanical ventilation cause further lung injury in ARDS patients
II. Spontaneous breathing efforts during mechanical ventilation are beneficial for patients with ARDS

*I. Spontaneous efforts during mechanical ventilation cause further lung damage in patients with ARDS*

Changes in the transpulmonary pressure is recognized as the key injurious factor in “ventilator-induced lung injury” (VILI). Transpulmonary pressure is the difference between the airway pressure and the pleural pressure and is generated by the mechanical power (stretching pressure) applied to the lungs [10, 11••]. In patients with SB efforts, the pleural pressure becomes negative during inspiration and may thus contribute to a higher transpulmonary pressure compared with that observed in patients with no spontaneous efforts. The greater changes in transpulmonary pressure will result in a proportional increase in tidal volumes and contribute to further lung injury [11••].

According to experimental data, SB may also increase transvascular pulmonary pressure (i.e. the difference between the intravascular pressure and the pressure outside the vessels) as well as pulmonary blood flow due to negative pleural pressure during SB. This may in turn exacerbate pulmonary oedema in the injured lung and contribute to VILI [11••].

Over recent years, experimental data have familiarised us with the “pendelluft” phenomenon [12]. It describes the movement of air within the injured lung. During mechanical ventilation with SB, air can move from the non-dependent to the dependent lung regions with no change in the tidal volume due to a more negative swing in the pleural pressure in the dependent lung. Even if patients are ventilated with a lung protective ventilation strategy, there is a risk of lung injury by overstretching the dependent lung during spontaneous efforts.

In caring for ARDS patients, clinicians use PEEP, with or without recruitment manoeuvres, to prevent collapse of the alveoli and to improve oxygenation. However, in patients with preserved SB, there is a risk of VILI in the presence of suboptimal PEEP [13]. In the secondary analysis of the LUNG SAFE study, data showed that clinicians used significantly lower PEEP in ARDS patients with preserved SB efforts than when ventilation was fully controlled, indicating that the risk is either discounted or not recognised [14••]. The data was collected from 459 ICUs in 50 countries and is to date the best available source of “real-life” evidence on how we treat our patients. This study is of great value for clinical researchers seeking to identify clinical practices at odds with evidence from clinical trials or practice variation due to a lack of relevant clinical trial data.

In a randomised controlled trial in paediatric ARDS patients, Ganesan et al. [15••] compared airway pressure release ventilation (APRV) and conventional low-tidal volume ventilation (LTV). The trial was terminated early due to an interim analysis showing increased mortality in the APRV group (53.8% vs. 26.9%). However, patients in the APRV group were younger (36 vs. 65.5 months) and had more severe ARDS than those in the LTV group. There was no information about any use of NMBA or monitoring of transpulmonary pressure, and the actual fractions of minute volumes from mandatory breathing to the total minute volumes were not calculated.

In critically ill patients, asynchronous ventilation is associated with prolonged mechanical ventilation and increased mortality [16]. Breath stacking is one of the most common forms of patient-ventilator asynchronies and happens when inspiratory efforts occur towards the end of the tidal volume delivered by the ventilator. This leads to a second breath before complete exhalation of the first breath. This is called double triggering. The resulting tidal volume will often exceed that determined by a lung protective LTV strategy and may worsen lung injury. A small observational trial by Pohlman et al. (*n* = 20, 85% of patients with ARDS) [17] reported frequent breath stacking in LTV that resulted in a median tidal volume of 10.1-mL/kg predicted body weight (1.62 times the set tidal volume).

*II. Spontaneous efforts during mechanical ventilation are beneficial for patients with ARDS*

Lightly sedated or awake patients may not tolerate controlled ventilation with small tidal volumes and ventilation in the prone position. Recent observational data suggest that a large proportion of ARDS patients are ventilated with techniques allowing SB, and that this is associated with more ventilator-free days without any negative impact on mortality [14••]. Thus, it seems that many clinicians believe that there is a reasonable trade-off in allowing light sedation when tolerated by the patient and, as a consequence, to allow spontaneous ventilatory efforts as well as larger tidal volumes than recommended.

Preventing ventilator-induced diaphragmatic dysfunction (VIDD) by allowing muscle contractions in the entire diaphragm is another reason to favour SB in ARDS patients. VIDD is the loss of diaphragmatic force-generating capacity specifically related to the use of mechanical ventilation [18]. Several experimental studies have shown that ventilation modes with some spontaneous effort may prevent VIDD [19, 20].

During spontaneous efforts, the movement of the entire diaphragm improves ventilation-perfusion (VQ) matching, recruits the lung, and improves oxygenation [11••]. An experimental study by Pellegrini et al. [21] showed that the diaphragm plays an important role in regulating expiration by preserving lung volume and protecting against lung collapse.

Reverse triggering, i.e. when contractions of the diaphragm are triggered by the ventilator (instead of the reverse), may occur even in the heavily sedated ARDS patient and may result in increased transpulmonary pressure and tidal volume. Recently, a multicentre observational study investigated the prevalence of reverse triggering and associated clinical outcomes during early ARDS [22]. The investigators included 100 patients, 92% of whom had mild or moderate ARDS. Fifty percent of patients exhibited reverse triggering, but most events (97%) were not associated with breath stacking. The probability of reverse triggering was associated with use of lower tidal volumes and less use of opiates. The data did not indicate any injurious effects of reverse triggering in this setting, and the combination of LTV, light sedation and early SB efforts was in fact associated with a reduction in 90-day mortality.

At the Critical Care Reviews meeting in Belfast, January 2020, Jean-Christophe M Richard (Lyon, France) presented unpublished results of the BiRDS multicentre randomised trial (NCT01862016, Early spontaneous breathing in acute respiratory distress syndrome) [23••]. Seven hundred and two patients with moderate or severe ARDS were randomised to either spontaneous ventilation with bilevel positive airway pressure (BIPAP)/APRV (*n* = 346) or to controlled ventilation (assist control ventilation) (*n* = 351). The target SB in the APRV group was between 10 and 50% of the minute volume permitted. The trial failed to show any difference in all-cause hospital mortality, and the authors concluded that SB in patients with ARDS is feasible and safe.

In another randomised trial comparing APRV (*n* = 71) and LTV (*n* = 67) in moderate and severe ARDS, there was reduced ICU mortality (19.7 vs. 34.3%, *p* = 0.053) and a higher median number of ventilator-free days in the APRV group. The driving pressures were similar in both groups [24].

## What About COVID-19 Associated ARDS?

The year 2020 has been dominated by the coronavirus disease 2019 (COVID-19) pandemic, and in several countries, there has been a huge burden on the healthcare system. ICUs worldwide suddenly had to deal with thousands of patients with COVID-19 associated ARDS. Based on observations and experience from several cases in the first wave of COVID-19 in Northern Italy, Gattinoni et al. [25] described COVID-19 associated ARDS as different from non-COVID ARDS. The authors described two phenotypes, type L for the early phase and type H for the late phase (best identified by CT scan). COVID-pneumonia type L is characterized by low elastance, low ventilation-perfusion (VQ) ratio, low lung weight and low recruitability, and type H is characterized by high elastance, high right to left shunt, high lung weight and high recruitability. The progression of the disease in type L patients depends on the severity of disease and SB which may result in patient self-inflicted lung injury (P-SILI). Over 50% of the observed cases were type L patients and 20–30% were type H patients, with the same features as in severe non-COVID ARDS. Based on these observations, the authors suggested different ventilator strategies: type L patients may be treated with higher tidal volumes and lower PEEP while type H patients should be treated as typical severe non-COVID ARDS [26].

Marini and Gattinoni [27] also described a treatment approach where in mild cases SB can be maintained in type L patients with oxygen or nasal high flow or non-invasive ventilation support as long as the patient does not exert excessive inspiratory efforts. According to these authors, tidal volume up to 7–8-ml/kg/ideal body weight is acceptable in this group of patients without increasing the risk of VILI. The purported benefits are to avoid reabsorption atelectasis and hypercapnia. Regarding SB in the weaning phase, the authors also recommend spontaneous breathing trials only at the end of a weaning phase, due to a risk of increased oedema, P-SILI and increased oxygen demand [27].

Recently, the above description has been challenged. In several observational cohorts, authors find that COVID-19 associated ARDS shares features with non-COVID ARDS, including compliance, driving pressures and plateau pressures [28–32]. As a consequence, it is advocated that the current best evidence for the management of non-COVID ARDS is valid also in COVID-19 associated ARDS and that the principles of lung protective mechanical ventilation should be applied to all ARDS patients regardless of aetiology [33].

## Do We Need Different Strategies According to the Severity of ARDS?

The harm that may result from early SB in ARDS is VILI due to increased transpulmonary pressure and tidal volumes. However, despite experimental findings, preliminary results from a clinical trial (BiRDS) and observational data (LUNG SAFE) indicate that patients can be managed with SB without undue harm. There is an urgent need to establish the safety of such an approach, as it may facilitate liberation from mechanical ventilation and reduce the need for sedatives, allowing patients to participate in early rehabilitation. This is probably relevant in all ARDS patients but will be easier to achieve in patients with mild or moderate ARDS.

The consequences of deep sedation in patients managed with mechanical ventilation were recently analysed in a systematic review and meta-analysis by Stephens et al. [34] and included nine studies with a total of 4521 patients published from 2012 to 2017. The authors concluded that deep sedation was associated with increased mortality and length of stay. Several studies have demonstrated that lung protective ventilation is feasible without deep sedation [35–37]. However, light sedation may be a challenge in some patients with severe disease and prone positioning.

The ROSE trial is a recently published randomised controlled trial investigating the benefits of early use of NMBA in patients with moderate to severe ARDS [36]. Patients were randomised to 48 h of continuous infusion of cisatracurium and deep sedation or to usual care with light sedation and no routine use of NMBA. The trial failed to show any difference in 90-day mortality and was stopped early due to futility after the second interim analysis. However, there were some serious adverse cardiovascular events in the intervention group (NMBA) compared with the usual care group. The results of the ROSE trial are at odds with findings in a previous trial (ACURASYS) [38], in which deeply sedated patients with moderate to severe ARDS appeared to profit from early neuromuscular blockade. Presently, NMBA is recommended only after conventional approaches with LTV, and prone ventilation has been attempted, and with a preference for intermittent rather than continuous dosing [36, 39, 40].

## Post-ARDS Recovery

The negative consequences of surviving an ICU stay have received increased attention in recent years. In general, more patients are being discharged alive from the ICU today than in previous decades [41]. The epidemiological literature on ARDS is complex, with controlled trials indicating improving survival, while large observational studies find hospital mortality to be firmly set at 40%, ranging from 35 to 46% according to initial severity category [2••, 42]. Survivorship may bring its own challenges for these patients, and there are potential health benefits in giving more attention to the recovery phase following ARDS [43–45]. The identification of the post-intensive care syndrome (PICS) has been of great importance for increasing awareness about health issues in survivors [46]. PICS conceptualises the direct effects of critical illness on physical, psychological and cognitive domains of health. Health challenges in more than two domains have been described in 21% of the survivors at 12 months post ICU [47]

The treatment approach selected for these patients during the ICU stay may have an important impact on long-term mental and physical outcomes. ARDS patients managed with strict controlled mechanical ventilation require deep sedation and occasionally NMBA. These are important risk factors for hospital-acquired delirium that may be modified. Up to 70–80% of ARDS cases are reported to be complicated by delirium [37]. It is well established that delirium increases the risk of worsening cognitive function and posttraumatic stress disorder, as well as increased post-discharge morbidity and mortality, hospital length of stay and costs [48]. Recently, a meta-analyses by Goldberg et al. [49] confirmed that delirium in surgical and non-surgical patients is associated with long-term cognitive decline. Importantly, in a quality improvement trial in ARDS patients managed with mechanical ventilation, Hager et al. [50] demonstrated that a reduction in sedative use was associated with more days awake without delirium but also that the proportion of days with delirium per patient increased (38% vs. 20%).

The prevalence of cognitive impairment in ARDS patients is high; 46% at 1 year and 25% at 6 years post-discharge [51]. Patients’ quality of life and psychosocial wellbeing are significantly reduced by cognitive impairment. This also affects caregivers as described by Sanfilippo et al. [52] who found that caregivers of ARDS survivors are at high psychological risk. At a median follow-up of 2.7 years, caregivers reported 39–52% risk of depression and 39% anxiety, and as many as 61% had signs of posttraumatic stress disorder. ARDS patients in this trial were managed with venovenous extracorporeal membrane oxygenation (VV-ECMO), and these patients were also at high risk of psychological impartment and reduced quality of life.

These findings address the importance and need for interdisciplinary post-ICU follow-up of both survivors and caregivers. Involvement of ICU clinicians in long-term follow-up may serve as a stimulus for changes in clinical practice necessary to mitigate long-term morbidity [53].

## Do We Need to Change Our Approach?

The evidence reviewed so far seems to favour SB during mechanical ventilation in some ARDS patients; there is little clinical evidence of harm and a possible benefit of light sedation and avoidance of NMBA in routine management. This may potentially translate into shortened time on mechanical ventilation, promote early mobilization and mitigate the long-term consequences of delirium. However, the available evidence may be biased, due to numerous unknown confounders, and a lack of definitive trial data continues to make this a contentious issue.

A sobering finding from the LUNG SAFE study is that clinical staff often does not recognize ARDS in patients with AHRF, and that the crude mortality rate of patients with ARDS remains at approximately 40% [54]. Some of this mortality is attributable to iatrogenic injury, and this calls for clinical researchers to investigate multiple strategies to mitigate the consequences of mechanical ventilation and prolonged ICU length of stay.

## Conclusion

In 2020, a new generation of ICU clinicians were faced with the challenge of caring for an unprecedented number of severely ill ARDS patients, sometimes using techniques that are at odds with existing guidelines, and with mixed results. Fortunately, much work has been published. We are presently undertaking a systematic scoping review of existing clinical data on SB with mechanical ventilation in patients with ARDS [55]. We believe that this will provide useful information that will help us determine how new trials may be designed to inform clinical practice.
